# Association Between Lupus Nephritis and Renal Clear-Cell Carcinoma: A Case Report and Review of the Literature

**DOI:** 10.7759/cureus.80459

**Published:** 2025-03-12

**Authors:** Thaís Fontenele da Ponte, Lysiane Maria Adeodato Ramos Fontenelle, Carlos Ewerton Maia Rodrigues, Jean Carlos Souza, Emanuelle de Matos Rodrigues

**Affiliations:** 1 Rheumatology, Fortaleza General Hospital, Fortaleza, BRA; 2 Medical Sciences, Medical School, University of Fortaleza, Fortaleza, BRA; 3 Rheumatology, Federal University of Ceará, Fortaleza, BRA

**Keywords:** general internal medicine, lupus nephritis, oncourology, renal cancer, systemic lupus erythematosus

## Abstract

Systemic lupus erythematosus (SLE) is an autoimmune disease affecting several organs, including the kidneys, potentially leading to lupus nephritis (LN). SLE has also been associated with several neoplasias, but its relation to renal cell carcinoma (RCC) has been little explored. We report a young women diagnosed concomitantly with LN and RCC. The latter was discovered incidentally during an investigation of nephrotic syndrome and confirmed on histology and renal microscopy. The patient was submitted to partial nephrectomy and immunosuppression, with good outcome, as shown by the improvement in proteinuria and other symptoms. Our case highlights the complexity of diagnosing SLE in combination with RCC and the importance of permanent surveillance and multidisciplinary approach to optimize treatment.

## Introduction

Systemic lupus erythematosus (SLE), an autoimmune disease with a relapsing and remitting course, causes the production of autoantibodies, complement activation, and the deposition of immune complexes, impacting several organ systems [1]. Lupus nephritis (LN), a common but severe complication of SLE, involves glomerular inflammation and can lead to chronic kidney failure, requiring treatment with immunosuppressants to prevent further complications [2].

However, exposure to immunosuppressants favors the development of neoplasia, a major cause of morbidity and mortality in SLE patients associated with a two- to five-fold increase in death risk [2]. In fact, the incidence of cancer, especially of the thyroid, cervix, and blood, is usually increased in this patient population [3].

Longitudinal studies, such as Tallbacka et al., have found SLE patients to have an increased risk of non-Hodgkin lymphoma and renal cancer [4]. The most common form of cancer in this scenario, renal cell carcinoma (RCC), has several subtypes, of which clear cell carcinoma is the most prevalent and most lethal [5].

In the diagnosis of nephritic and nephrotic syndromes, the several possible causes of glomerulonephritis (GN), such as those related to SLE or paraneoplastic syndrome, must be carefully distinguished, especially when nephritis occurs concomitantly with cancer but without a preexisting immune disease [6]. This distinction is crucial for correct diagnosis and adequate treatment.

In this paper, we describe a patient diagnosed concomitantly with LN and RCC, which were confirmed on histology. The prognosis was good following nephrectomy and immunosuppression. We also reviewed the literature on this rare combination.

This descriptive, cross-sectional, and retrospective study analyzed a female patient with concurrent LN and RCC treated at a referral hospital in Northeastern Brazil. Data were retrieved from medical records, and a literature review was conducted using PubMed, Science Direct, SciELO, and Google Scholar with specific search terms.

## Case presentation

A 32-year-old female patient with no previous comorbidities was admitted to our service (Fortaleza General Hospital) on June 14, 2023, at 2:00 p.m. One month earlier, she had developed erythematous and bullous lesions in photoexposed areas, oral ulcers, malar rash, alopecia, polyarthritis, and lower limb edema. This was followed by anasarca, resting dyspnea, renal dysfunction, and increased C-reactive protein levels and erythrocyte sedimentation rate, leading to her transfer from the hospital in her hometown for further investigation.

While hospitalized, the patient’s diagnostic evaluations revealed an antinuclear antibody titer of 1/640, with a fine speckled pattern, anti-DNAds positivity, hypocomplementemia, bilateral pleural effusion, hematuria and proteinuria in the urine analysis, and 24-hour proteinuria of 11 g (Table 1). These findings confirmed the suspicion of SLE, as per the 2019 European Alliance of Associations for Rheumatology/American College of Rheumatology criteria [7].

**Table 1:** Results of complementary tests during follow-up CRP: C-reactive protein; ESR: erythrocyte sedimentation rate; LDH: lactate dehydrogenase; RBC: red blood cells; ANA: antinuclear antibody; SSA: Sjögren's-syndrome-related antigen A; SSB: Sjögren's-syndrome-related antigen B; HCV: hepatitis C; HbsAg: hepatitis B surface antigen; VDRL: Venereal Disease Research Laboratory

Variable	June 2023	August-September 2023	September 2024	Reference value
Hemoglobin	9.1 g/dL	12.1 g/dL	12 g/dL	13.5-17.0 g/dL
Leukocytes	9,100/mm^3^	6,500/mm^3^	9,280/mm^3^	3,600-11,000/mm^3^
Neutrophils	5,040/mm^3^	3,958/mm^3^	6,245/mm^3^	1,440-8,360/mm^3^
Lymphocytes	2,230/mm^3^	2,020/mm^3^	2,143/mm^3^	720-4,400/mm^3^
Platelets	303,000/mm^3^	406,000/mm^3^	364,000/mm^3^	150,000-450,000/mm^3^
Urea	58 mg/dL	36 mg/dL	30 mg/dL	10-50 mg/dL
Creatinine	1.4 mg/dL	0.84 mg/dL	0.76 mg/dL	0.6-1.1 mg/dL
TGO	26 mg/dL	-	-	Up to 32 mg/dL
TGP	24 mg/dL	-	-	Up to 31 mg/dL
Bilirubin total	0.16 mg/dL	-	-	Up to 1.0 mg/dL
CRP	39.4 mg/L	6.8 mg/L	-	Up to 3.0 mg/L
ESR	51 mm	60 mm		Up to 10 mm
Reticulocytes	49,300/mm^3^	-	-	50,000-100,000/mm^3^
Direct Coombs	Positive	-	-	Negative
LDH	246 U/L	-	-	125-250 U/L
Haptoglobin	105 mg/dL	-	-	30-200 mg/dL
ANA	1/640 fine speckled pattern	-	-	Negative
Anti-Sm	Negative	-	-	Negative
Anti-DNAds	Reactive	-	Not reactive	Not reactive
Anti-SSA	Not reactive	-	-	Not reactive
Anti-SSB	Not reactive	-	-	Not reactive
C3	45.3 mg/dL	95 mg/dL	122 mg/dL	90-180 mg/dL
C4	5.9 mg/dL	12.6 mg/dL	11 mg/dL	10-40 mg/dL
Lupus anticoagulant	Negative	-	-	Negative
Anticardiolipin IgM/IgG	Negative/negative	-	-	Negative/negative
Albumin	1.6 g/dL	2.9 g/dL	-	3.5-5 g/dL
Urine analysis	Proteins 3+, 20 RBCs/field, 15 leukocytes	Proteins 4+, 10 RBCs/field, 3 leukocytes	No proteins, no RBCs	No proteins, 0-3 RBCs/field, 0-5 leukocytes
24-hour proteinuria	11 g	1.5 g	0.26 g	Up to 300 mg
Anti-HIV	Not reactive	-	-	Not reactive
Anti-HCV	Not reactive	-	-	Not reactive
HbsAg	Not reactive	-	-	Not reactive
VDRL	Not reactive	-	-	Not reactive

**Figure 1:**
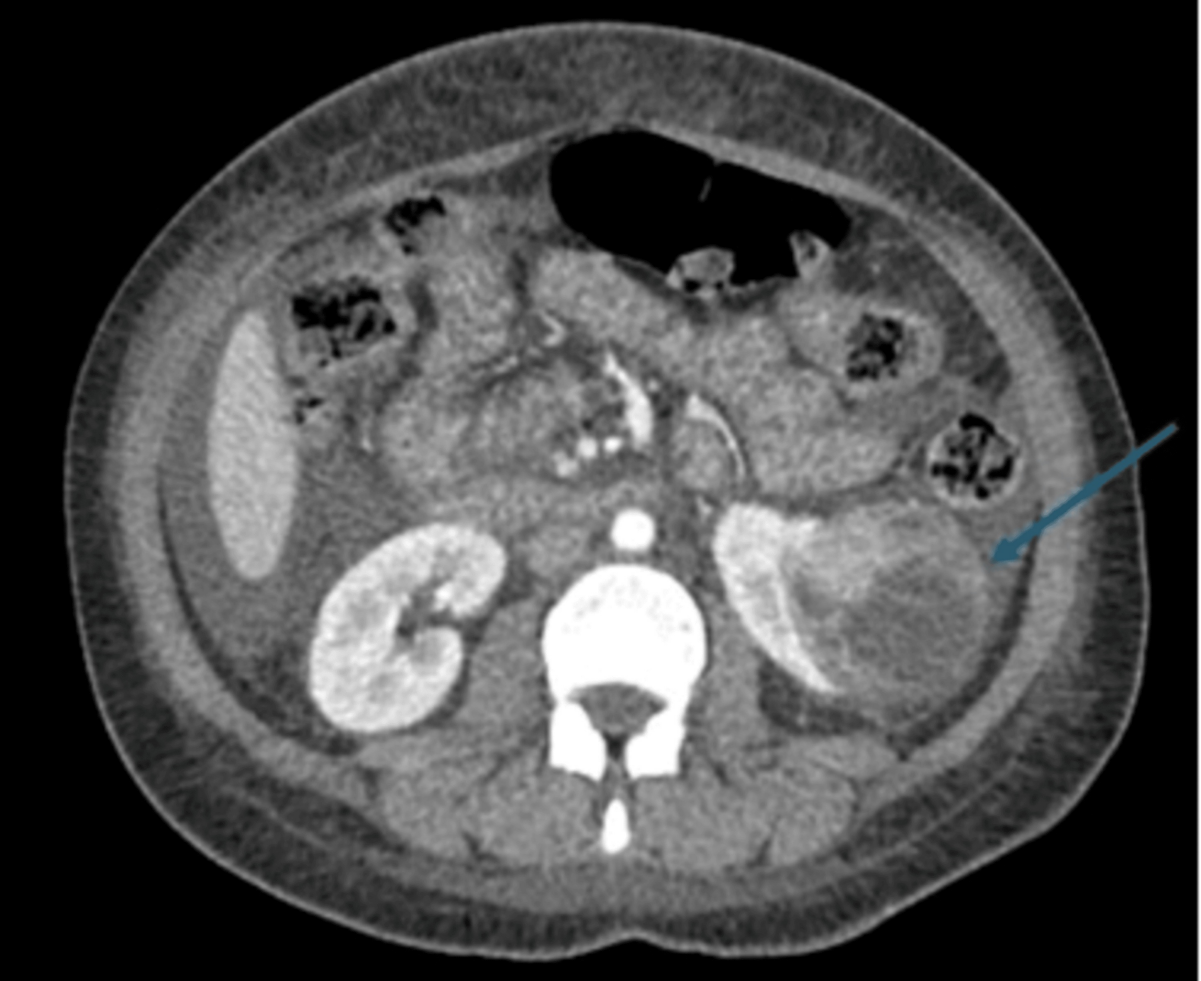
Axial section of abdominal tomography with contrast dye. The blue arrow indicates a heterogeneous solid cystic nodule in the lower third of the left kidney measuring 5.6 × 6.3 × 6.5 cm

The patient was given pulse therapy with methylprednisolone (500 mg/day for three days) and a dose of cyclophosphamide (CYC) (Euro-Lupus regimen) for LN. At this point, when preparing for the renal biopsy, the urinary system was ultrasound scanned, incidentally revealing an expansive lesion in the left kidney. This was confirmed on abdominal tomography with contrast dye, identifying a solid cystic nodule in the lower third of the left kidney measuring 5.6 x 6.3 x 6.5 cm, with heterogeneous contrast enhancement (Figure 1). Tomography scans of the chest and cranium for staging revealed no changes.

**Figure 2:**
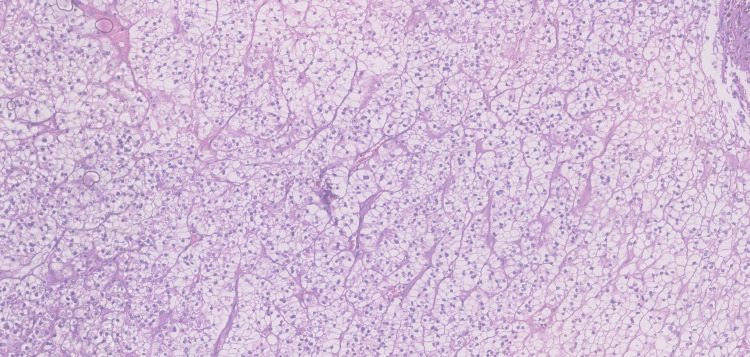
Malignant epithelial neoplasia consisting of clear cytoplasmic cells in a solid sheet-like pattern (hematoxylin and eosin stained)

**Figure 3:**
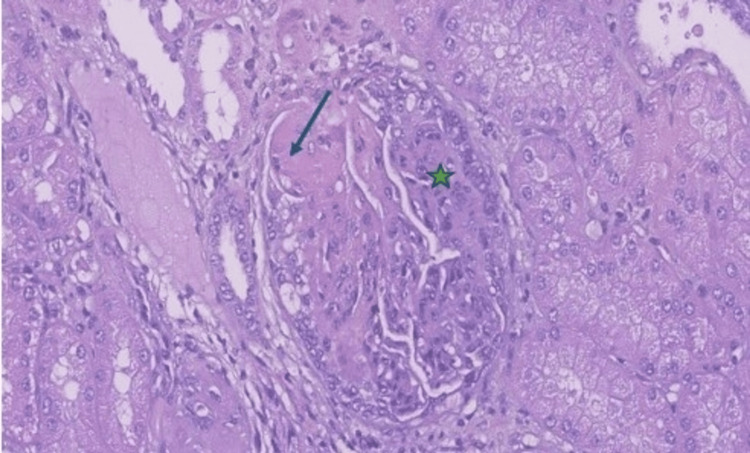
Glomerule with inflammation. The blue arrow indicates a subendothelial deposit with loop occlusion (pseudothrombus). The green star shows growing extracapillary cell proliferation compressing glomerular capillary tufts (hematoxylin and eosin stained)

**Figure 4:**
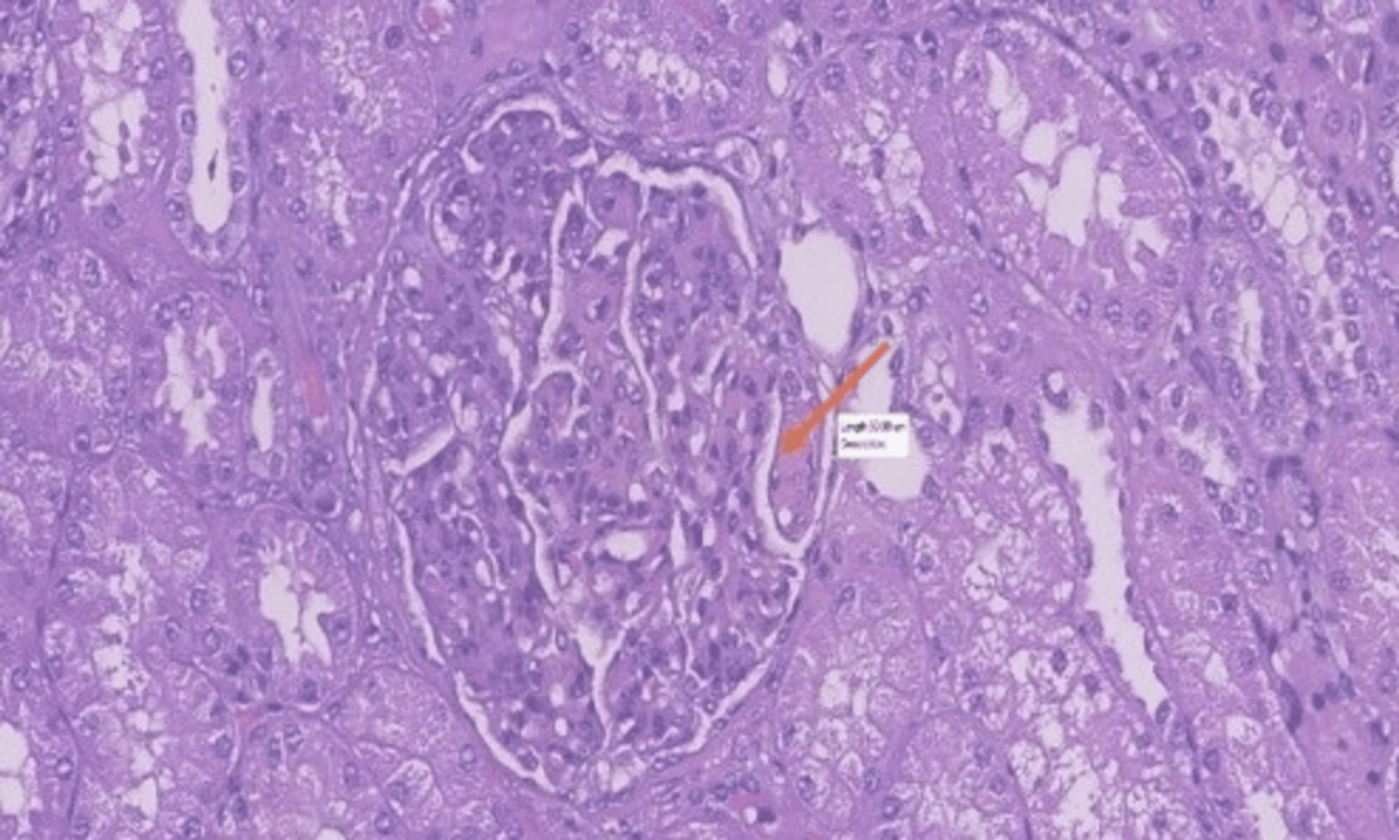
Enlarged glomerule with endocapillary hypercellularity and mixed leukocyte infiltrate. The orange arrow indicates an intravascular deposit (hematoxylin and eosin stained)

In July 2023, the patient was submitted to partial nephrectomy, leading to the concomitant diagnosis of LN and RCC. Histopathology and renal optic microscopy confirmed clear-cell carcinoma restricted to the kidney, with free margins, in addition to class IV LN, without inflammation or interstitial fibrosis, an activity index of 11/24, and a chronicity index of 1/12 (Figures 2-4). Surgery was considered curative for cancer staged as T1bNxM0-stage I [8], characterized by early-stage cancer, with the tumor confined to the kidney, no evidence of lymph node involvement, and no signs of metastasis. Following surgery, 24-hour proteinuria and edemigenic syndrome improved significantly.

**Figure 5:**
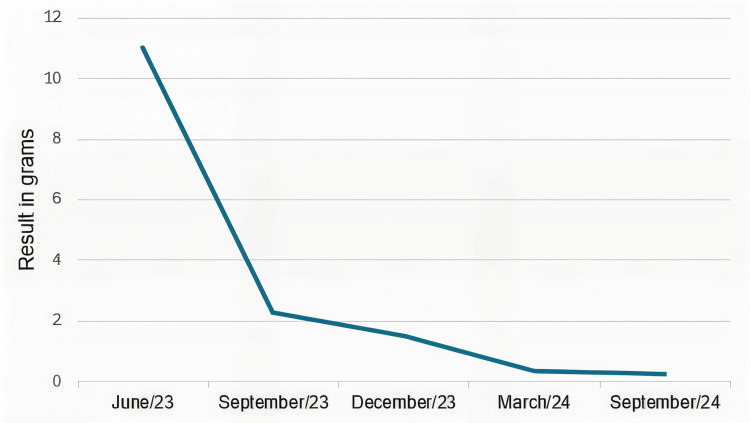
Evolution of 24-hour proteinuria during follow-up

In September 2023, during a follow-up at the rheumatology service, treatment for SLE was restarted, replacing CYC with mycophenolate mofetil (MMF) (3 g/day) and hydroxychloroquine (5 mg/kg/day) and withdrawing the glucocorticoid. The patient is currently in remission of LN, with a significant decrease in 24-hour proteinuria (Figure 5), and maintaining a reduced and stable regimen of immunosuppression, with no steroids. The neoplasia did not recur, and the patient continues with multidisciplinary follow-up.

## Discussion

We report a rare case of LN and renal clear-cell carcinoma diagnosed concomitantly. Treatment was challenging due to the classic SLE symptoms, with renal involvement requiring immunosuppression. Cancer symptoms are sometimes masked by SLE activity. In our patient, neoplasia was diagnosed incidentally based on changes observed on ultrasonography.

RCC accounts for approximately 2% of cancer diagnoses and deaths worldwide, with a higher prevalence in men aged 60 years [5]. Our patient, who is young and female, was atypical, and the RCC was likely related to SLE. The main risk factors for RCC, which include obesity, hypertension, and smoking, were not present in our patient.

SLE has been positively associated with cancer risk in both sexes. Several studies have suggested a connection with specific types of cancer, such as renal cancer [2] or (perhaps less evidently) cancer of the breast, ovaries, endometrium, and skin [9].

**Figure 6:**
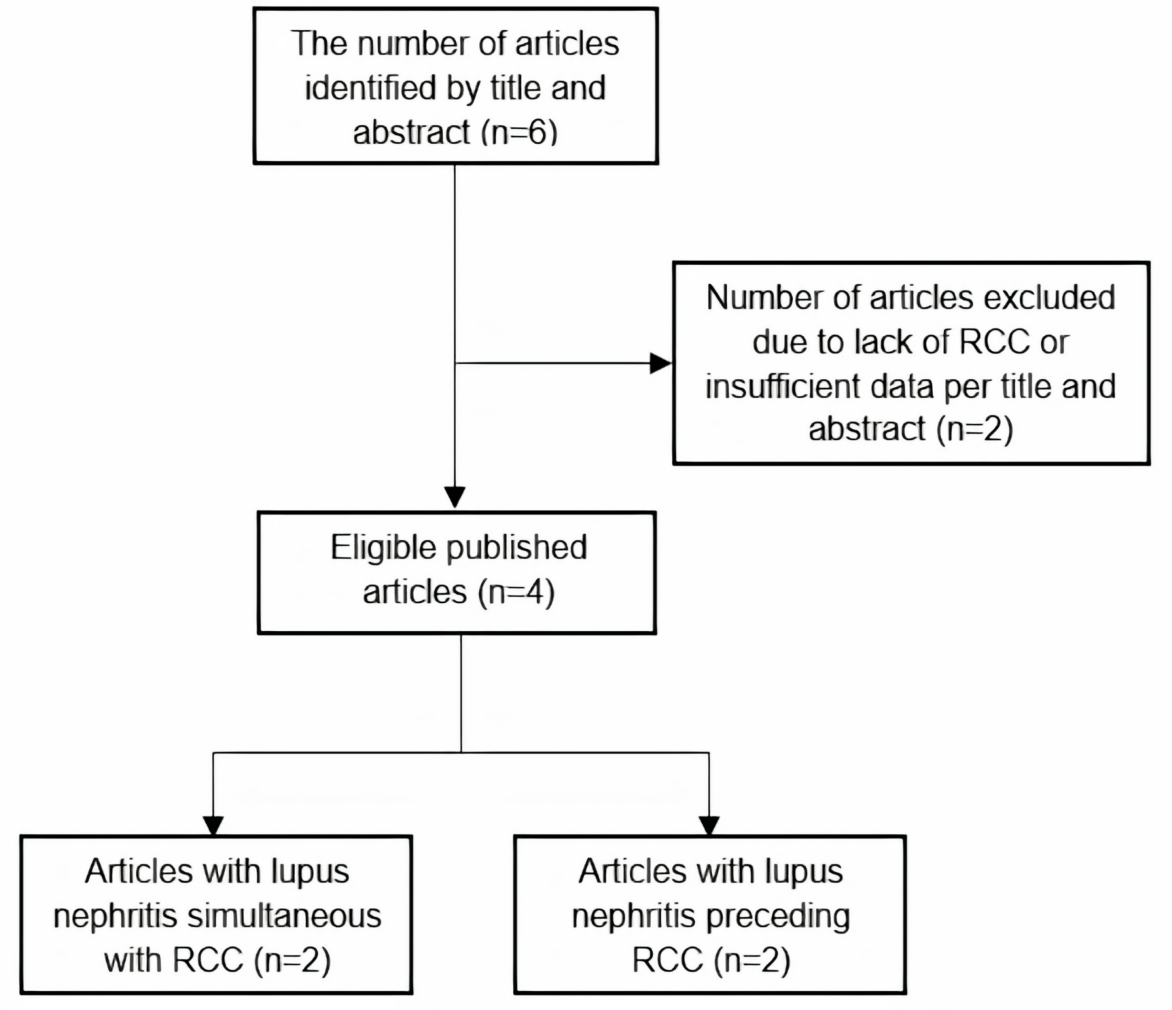
Flowchart of selection of published cases of LN and RCC LN: lupus nephritis; RCC: renal cell carcinoma

Initially, six publications were identified, two of which were excluded (one case had a nonneoplastic renal mass and the other had insufficient data) (Figure 6). Thus, the final review covered four SLE patients diagnosed with LN and RCC [10-13] published between 1996 and 2022. The clinical and histopathological findings of these cases, along with their respective outcomes, are presented in Table 2.

**Table 2:** Reviewed cases of combined LN and RCC AZA: azathioprine; MP: methylprednisolone; CYC: cyclophosphamide; NIH: National Institutes of Health; CKF: chronic kidney failure; MMF: mycophenolate mofetil; LN: lupus nephritis; RCC: renal cell carcinoma; SLE: systemic lupus erythematosus

Study	Patient profile	Stage/RCC subtype	Diagnosis of LN and RCC concomitant	Class/treatment for LN	Clinical outcome
Wong [10]	Female, 52 years, SLE for one year	I/chromophobe	No (LN for one year)	III/CYC (NIH regimen) (total dose: 6 g)	Nephrectomy; maintenance w/MMF; no recurrence of RCC in six months
Mehta and Gandhi [11]	Male, 43 years	I/chromophobe	Yes	IV/CYC (Euro-Lupus regimen) (total dose: 3 g)	Partial nephrectomy; maintenance w/MMF; proteinuria improved
Matsuda et al. [12]	Female, 44 years, SLE for 26 years	I/clear-cell	Yes	IV/pulse therapy w/MP + CYC (dose not informed)	Nephrectomy; corticoids; CKF
Gopalakrishnan et al. [13]	Female, 34 years, SLE for eight years	I/clear-cell	No (LN for eight years)	II/AZA and prednisone	Partial nephrectomy; AZA; no recurrence of RCC in two years

The reviewed cases were aged 34-52 years, and three were female (75%), matching the profile of SLE patients in general. In three cases, SLE was diagnosed before RCC (1-26 years). As in the case reported here, Mehta and Gandhi diagnosed LN and RCC concomitantly [11]. Three reviewed cases used immunosuppressants (CYC and azathioprine) to treat nephritis [10,11,13].

In most cases, RCC was detected incidentally on ultrasonography or tomography. Wong diagnosed an LN patient with RCC when investigating persistent hematuria despite treatment with steroids, CYC, and MMF [10]. It was suggested that RCC may have been present before LN was diagnosed, resembling our own patient.

The RCC subtypes varied between the studies. Two reports identified the clear-cell subtype, as in our case. In all cases, RCC was stage I, based on renal cancer TNM staging (eighth edition), justifying the prescription of nephrectomy [8]. No patient developed metastases during the period covered by the review, and all received immunosuppressants for SLE when necessary.

The relationship between SLE and cancer risk is not well understood, but the chronic multiorgan inflammation typical of SLE favors tissue damage and cell apoptosis, both of which are involved in the development of malignancies [9]. Age, genetic predisposition, and environmental triggers are also significant [5].

The interferon-γ-stimulated secretion of cytokines like CXCL10 is a key mechanism in autoimmune diseases (including SLE) perpetuating inflammation and autoimmunity, potentially favoring carcinogenesis [14]. Also, several costimulatory molecules (e.g., CTLA4) play a crucial role in the pathogenesis of both SLE and cancer, suggesting a level of interconnectedness between these two conditions [15].

Immunosuppressants like AZA have been associated with an increased risk of cervical dysplasia and cancer [16]. One of the reviewed cases (Table 2) used medication for LN and developed RCC, but no direct relationship was established between the neoplasia and the drug, and the medication was maintained [13]. On the other hand, when used in autoimmune diseases and after transplantation, MMF has not been found to increase the risk of lymphoma or other malignancies in prospective studies with postrenal-transplant patients [17].

CYC can suppress immunological surveillance and induce cytotoxic effects, favoring mutagenesis. According to some authors, CYC can increase the risk of cancer years after treatment suspension, depending on the cumulative dose [18]. In the case reported here, the low dose of immunosuppressant and the concomitant diagnosis make a connection between the medication and neoplasia unlikely.

GN has been associated with solid and hematological malignancies. Among the glomerular diseases associated with cancer, membranous nephropathy and minimal change disease are the most common [6]. Ryu et al. found that, regardless of etiology, GN patients over 50 years of age are at greater risk of cancer than the general population, highlighting the importance of screening in this age group [19].

RCC is believed to be associated with paraneoplastic syndromes in 10%-40% of cases, usually along with symptoms like hypercalcemia, fever, and erythrocytosis. Additionally, glomerulopathy can manifest as part of this syndrome [20]. However, our patient did not present these laboratory alterations. Despite improvements in proteinuria after the treatment, renal microscopy confirmed the diagnosis of LN, supporting this conclusion.

Historically, RCC was diagnosed with the classic triad of flank pain, macroscopic hematuria, and palpable abdominal mass [5]. However, most cases are now detected incidentally due to the increase in the use of abdominal imaging, allowing the identification of small, localized tumors. In fact, our own patient was diagnosed on occasion of radiological scanning, with no evidence of metastases.

Treating patients with SLE and cancer is challenging as immunosuppression aimed at controlling SLE can compromise response to cancer, facilitate infections, and shorten survival. Renal clear-cell carcinoma, even after curative nephrectomy, leads to metastases in 30% of cases, requiring systemic therapy and making management more complex [20].

No specific strategies of neoplastic screening exist for SLE patients, apart from general guidelines [3]. Universal screening for RCC is not cost-effective due to the low incidence of the condition [5]. Follow-up of RCC patients in the first two years should include chest and abdominal tomography at intervals of three to six months (high risk) or 12 months (low risk), though the long-term benefits of this practice are uncertain [20].

## Conclusions

The relationship between SLE and RCC is complex and little explored, and the concomitant diagnosis of LN and renal clear-cell carcinoma is rare. Our study stresses the importance of adequate treatment for a better prognosis and reduced morbidity and mortality. Screening for cancer in SLE patients remains controversial and should be discussed with the individual patient at the rheumatology service.
